# A Multidisciplinary Approach to Unplanned Conversion from Off-Pump to On-Pump Beating Heart Coronary Artery Revascularization in Patients with Compromised Left Ventricular Function

**DOI:** 10.1155/2014/348021

**Published:** 2014-11-12

**Authors:** Georgia Tsaousi, Antonis A. Pitsis, George D. Ioannidis, Dimitrios G. Vasilakos

**Affiliations:** ^1^Department of Anesthesiology and ICU, Faculty of Medicine, Aristotle University of Thessaloniki, Stilponos Kiriakidi 1, 54636 Thessaloniki, Greece; ^2^Department of Cardiosurgery and ICU, Saint Luke's Clinic, Panorama, 55236 Thessaloniki, Greece

## Abstract

*Aim*. To comparably assess the perioperative risk factors that differentiate off-pump coronary artery bypass (OPCAB) grafting cases from those sustaining unplanned conversion to on-pump beating heart (ONCAB/BH) approach, in patients with left ventricular ejection fraction (LVEF) < 40%. *Methods*. Perioperative variables were retrospectively assessed in 216 patients with LVEF < 40%, who underwent myocardial revascularization with OPCAB (*n* = 171) or ONCAB/BH (*n* = 45) approach. The study endpoints were operative mortality (30-day) and morbidity assessed by length of intensive care unit stay (LOS-ICU), using 2 days as cut-off point. *Results*. Poor LVEF, increased EuroSCORE II, acute presentation, congestive heart failure, cerebrovascular disease, perioperative renal impairment, clinical status deterioration upon admission and during ICU stay, acute myocardial infarction, and low cardiac output syndrome supported by inotropes and/or balloon-pump counterpulsation were significantly related to ONCAB/BH group (*P* < 0.05). EuroSCORE II (*P* = 0.01) and LVEF (*P* = 0.03) were the most powerful discriminative predictors of intraoperative conversion to ONCAB/BH. Operative mortality was 2.9% in OPCAB and 6.6% in ONCAB/BH group (*P* = 0.224), while 23.4% participants in OPCAB and 42.2% in ONCAB/BH approach had a LOS-ICU > 2 days (*P* = 0.007). *Conclusions*. Patients with LVEF < 40% undergoing ONCAB/BH are subjected to more preoperative comorbidities and implicated ICU stay than their OPCAB counterparts, which influences adversely short-term morbidity, while operative mortality remains unaffected.

## 1. Introduction

Advanced left ventricular (LV) dysfunction from coronary artery disease constitutes an intriguing clinical entity in patients undergoing CABG [1–3]. In this subpopulation, myocardial revascularization is often performed to ameliorate ischemic symptoms, improve LV performance, and prevent future ischemic events [1, 2]. Albeit, in symptomatic multivessel disease with concomitant limited cardiac reserve, conventional cardiopulmonary bypass surgery (ONCAB) is considered as the optimal approach, it has been speculated that extracorporeal circulation (CPB) in this surgical approach could exacerbate myocardial damage in patients with compromised left ventricle [2].

In an attempt to minimize the potential deleterious effects of CPB, off-pump CABG (OPCAB) has emerged as an attractive alternative to accomplish myocardial revascularization [3, 4]. Nevertheless, poor LV function is sometimes an exclusion criterion for the OPCAB approach because of the technical difficulty of safely displacing the heart under conditions of hemodynamic stability [5]. Under these circumstances, the current clinical practice offers the ability of performing CABG with CPB with an empty beating heart (ONCAB/BH), which is advantageous over ONCAB as it benefits from the hemodynamic stability afforded by CPB, without being implicated by cardioplegic arrest and possible myocardial ischemic damage [6].

Numerous reports have catalogued the risk factors predictive of morbidity and mortality in patients with limited cardiac reserve undergoing either on-pump or off-pump CABG, but there is limited knowledge regarding a thorough comparable risk analysis of perioperative variables between the two alternatives of coronary artery bypass grafting, in the current beating heart era [3, 5, 6].

Thus, we embarked on this study with a view to identify the contemporary pre-, per-, and postoperative risk factors that discriminate the cases undergoing OPCAB as planned revascularization strategy from those sustaining unplanned intraoperative conversion to ONCAB/BH, in a defined subset of patients with compromised LV function, in our institution. Furthermore, an assessment was made of the impact of both techniques on the operative mortality and length of intensive care unit stay (ICU), used as an index of short-term morbidity.

## 2. Material and Methods

### 2.1. Study Population

This study was conducted according to the guidelines laid down in the Declaration of Helsinki and all procedures involving human subjects/patients. After the Institutional Review Board approval of the study protocol and written informed consent have been obtained from each participant, the records of all consecutive adult patients with depressed LV ejection fraction (LVEF less than 40%), admitted in our postcardiac surgery ICU following isolated multivessel revascularization with the beating heart approach over a 28-month period (August 2010 to November 2012), were retrospectively analyzed. LVEF was assessed from echocardiography or cardiac catheterization, or both. In cases of maintaining hemodynamic stability during heart positioning to visualize target vessels, the OPCAB approach was applied; otherwise ONCAB/BH was instituted.

### 2.2. Clinical Data Collection and Definitions

Independent variables that might affect patient outcome were categorized as patient demographics, preexisting comorbidities, measurements of physiologic reserve, and postoperative complications. Demographics included common variables, such as age and sex. Data was collected on preexisting health problems, such as hypertension (under medical therapy for at least 2 years), diabetes mellitus, symptomatic cerebral or peripheral vascular disease, chronic obstructive pulmonary disease (COPD; under long-term bronchodilator therapy and/or a forced expiratory volume in 1 second < 75% of the predicted), and chronic renal dysfunction (defined as preoperative serum creatinine > 1.4 mg/dL) [2]. The number of diseased arteries was based on whether the right coronary artery, left anterior descending artery, or circumflex vessel in their major segments or major branches contained lesions greater than or equal to 50% in diameter or there was left main stem disease > 50%. Markers of altered physiologic reserve available before surgery included the degree of LV impairment, documented by the history of congestive heart failure and LVEF. Furthermore, New York Heart Association classification (NYHA class) and European System for Cardiac Operative Risk Evaluation II model (EuroSCORE II) were assessed preoperatively in every participant [7]. Acute presentation was defined as surgery within 24 hours of angiography, due to onset of acute coronary syndrome, unstable angina, signs of myocardial infarction, and/or severe myocardial dysfunction.

Furthermore, ICU outcome stratification algorithms, such as Acute Physiology and Chronic Health Evaluation (APACHE) II and System Organ Failure Assessment (SOFA) score, were assessed for each participant [8, 9]. The APACHE II score was calculated precisely using the worst values for the first 24 hours following ICU admission. The SOFA score was computed daily, starting on the first postoperative day until ICU discharge. In the calculation of the score, the worst values for each organ system in each 24-hour period were considered. In the present study we applied the maximum SOFA (MaxSOFA), defined as the maximum total score recorded in a single day during ICU stay [9].

Postoperative complications included development of low cardiac output syndrome (LCOS), reoperation (due to bleeding, suspected pericardial tamponade, or graft occlusion), postoperative acute myocardial infarction (AMI), acute kidney injury (AKI; defined as an increase in serum creatinine ≥ 0.3 mg/dL or ≥ 50% combined to urine output deterioration < 0.5 mL/kg/h for more than 6 hours), respiratory complications (ventilation failure, infection, reintubation, and tracheostomy), central nervous system (CNS) dysfunction (transient or permanent neurological deficit), and sustained supraventricular or ventricular arrythmias (atrial fibrillation, premature ventricular complexes, ventricular tachycardia, and fibrillation, documented by electrocardiographic monitoring and requiring treatment with medication or cardioversion). LCOS was defined as a cardiac index less than 2 L/min/m^2^ for at least 3 hours, despite optimal filling pressures (wedge pressure > 12 mmHg), being treated with inotropic drugs and/or intraaortic balloon counterpulsation (IABP) [8].

The analysis also included the number of grafts, the duration of mechanical ventilation, and ICU stay. Length of ICU stay more than two days was considered as the cut-off point to assess prolonged ICU stay. Operative mortality included all deaths within 30 days after the index operation irrespective of where the death occurred.

### 2.3. Statistical Analysis

Normality of data was assessed by Kolmogorov-Smirnov test. Univariate analysis was undertaken using Student's *t*-test for comparison of means of continuous variables and normal distributed data, while the Mann-Whitney rank sum test was used to compare means in the case of nonnormally distributed data. Subgroup comparisons of binomial data were assessed by a chi-square or Fisher's exact test for expected cell counts less than 5. Odds ratios with 95% confidence intervals were computed using a multivariate logistic regression model with conversion to ONCAB/BH as the response variable. Operative survival among the studied off-pump approaches was evaluated by Kaplan-Meier method. For all statistical procedures, a *P* value of less than 0.05 was considered significant. Data were analyzed using SPSS version 18.0 (SPSS Inc., Chicago, IL, USA).

## 3. Results

During the study period, a total of 809 patients were scheduled to undergo isolated myocardial revascularization via the OPCAB technique. Of the 216 patients with LVEF < 40% who were scheduled to undergo CABG with the off-pump technique and were admitted to our postcardiac surgery ICU, unplanned conversion to ONCAB/BH occurred in 45 (20.8%). This was attributed to hemodynamic instability, which could be expected given the decreased tolerance for positioning required by the OPCAB procedure. No case of intraoperative death occurred.

The age of the study group ranged from 40 to 79 (mean 64.1 ± 8) years. The sample constituted 177 men (82%) and 39 women (18%). Among them, 175 (81.3%) presented with a LVEF more than 25%, whereas 41 (18.7%) patients had a LVEF equal to or lower than 25%. The mean LVEF, NYHA class, and EuroSCORE II were 32.3 ± 7.1%, 3.1 ± 0.7, and 2.7 ± 3.4, respectively.

Baseline and peroperative clinical characteristics among the two subgroups are listed in Table 1. Univariate analysis identified severely deteriorated LVEF (*P* = 0.000), poor clinical status as reflected by the EuroSCORE II (*P* = 0.000), symptoms of congestive heart failure (NYHA class III/IV; *P* = 0.001), acute presentation for surgery (*P* = 0.000), preoperative renal impairment (*P* = 0.001), and cerebrovascular disease (*P* = 0.006), as common preoperative clinical features differentiating the participants among the two beating heart surgical techniques.

Furthermore, in our cohort, 81.7% (*n* = 176) of the patients presented with left main coronary artery disease. The average number of coronary bypass grafts per patient in the OPCAB group was comparable (*P* = 0.171) with the ONCAB/BH one (2.2 ± 0.9 and 2.4 ± 0.7, resp.).

The distributions of postoperative variables according to OPCAB and ONCAB/BH groups are reported in Table 2. The higher severity of acute illness on admission to ICU as reflected by the APACHE II score (*P* = 0.001), more implicated ICU stay evaluated by MaxSOFA score (*P* = 0.041), presence of LCOS (*P* = 0.013), need for inotropic support (*P* = 0.025) or IABP (*P* = 0.017), and development of AMI (*P* = 0.025) or AKI (*P* = 0.019) constituted the postoperative factors related to ONCAB/BH group in an important manner.

The duration of mechanical ventilation support for the total cohort ranged from 0.3 to 32 (median 0.7) days. The length of ICU stay ranged from 2 to 51 (median 2) days. Prolongation of ICU stay for more than 2 days was recorded in a total of 59 (27.3%) patients. Among them, 40 (23.4%) participants in OPCAB and 19 (42.2%) in ONCAB/BH technique were included (*P* = 0.007).

The application of stepwise logistic regression analysis on the studied preoperative risk variables identified EuroSCORE II (*β*, 0.385; SE, 0.154; OR, 1.470; 95% CI, 1.086 to 1.989; *P* = 0.01) and LVEF (*β*, 1.202; SE, 0.559; OR, 3.329; 95% CI, 1.112 to 9.963; *P* = 0.03) as the most powerful discriminative predictors of intraoperative conversion to ONCAB/BH technique.

**Figure 1:**
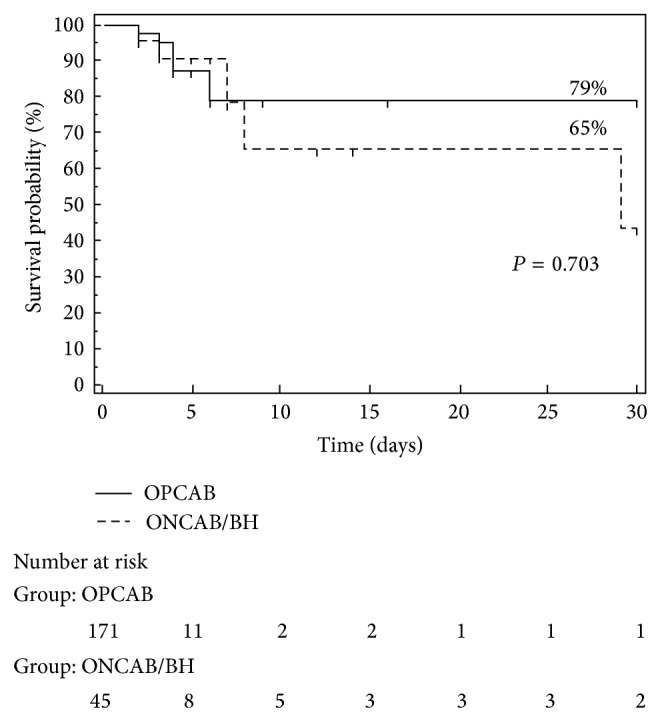
Kaplan-Meier curves for operational survival. Comparison of operational survival between patients undergoing OPCAB and those undergoing ONCAB/BH technique. *P* value calculated by the log-rank test was 0.703.

Operative mortality in the total cohort was 3.7% (8 patients). The ONCAB/BH presented a slightly increased mortality compared with the OPCAB group (6.6% versus 2.9%) but it failed to reach statistical significance (*P* = 0.224). The cause of 30-day death was ventricular arrythmias in 50% (*n* = 4), cardiogenic shock in 37.5% (*n* = 3), and irreversible neurological damage in 12.5% (*n* = 1). In terms of operative mortality, the survival analysis confirmed that ONCAB/BH and OPCAB groups presented comparable survival (HR, 0.813; 95% CI, 0.253 to 2.525; *P* = 0.703). The overall survival curves of ONCAB/BH and OPCAB patients are presented in Figure 1.

## 4. Discussion

The current trial suggests that the severely deteriorated LVEF and poor clinical status assessed by the EuroSCORE II could serve as the best discriminative predictors of unplanned intraoperative conversion from off-pump to on-pump beating heart technique in a defined subset of patients with depressed LV function, undergoing isolated surgical revascularization. ONCAB/BH approach is characterized by higher severity of acute illness upon admission and during the course of ICU stay evaluated by APACHE II and MaxSOFA score, respectively, development of LCOS, and acute cardiac events or renal impairment. Albeit operative mortality is comparable among the two beating heart techniques, short-term morbidity of patients undergoing ONCAB/BH revascularization seems to be affected in a rather unfavorable manner.

Despite the fact that multiple reports document increased operative and postoperative hazard for patients with deteriorated LV function due to limited physiological reserve, evolution of improved cardiac surgical techniques led to effectively restore nutrient blood flow to areas of ischemic myocardium with lasting effects aiming to ameliorate survival in this high risk subset of cardiosurgical patients [1–3, 5]. Nevertheless, suboptimal outcomes in this subset of patients have been attributed to the damaging effect of CPB on the myocardium. As techniques for surgical revascularization evolved, off-pump approach gained a foothold as a safe alternative to on-pump coronary artery surgery, and data began to emerge citing the potential benefits of avoidance of CPB in the high risk subset of patients with ischemic cardiomyopathy and a low LVEF [3]. Albeit OPCAB is an appealing technique regarding the better preservation of blood supply to the subendocardium and interventricular septal contractility, hemodynamic upheavals during heart dislocation could be implicated by serious hemodynamic deterioration entailing urgent transfer to conventional CPB [3, 4, 6]. In such cases, the reported outcome is rather disappointing [9].

An intermediary approach based on maintenance of a beating heart with CPB support but without aortic cross-clamping has been suggested as a feasible trade-off between conventional cardioplegia and off-pump operations [6, 9–11]. The absence of cardioplegic arrest coupled with the hemodynamic stability guaranteed during extensive heart manipulation is the biggest benefits coming from this technique, especially in patients with limited cardiac reserve.

Although several contemporary studies addressed primarily predictors of morbidity and mortality following either OPCAB or ONCAB/BH revascularization in patients with compromised LVEF, the perioperative factors differentiating the two alternatives of beating heart myocardial revascularization in this defined subset of patients are poorly investigated [3, 9, 12, 13].

In a clinical setting similar to ours, Darwazah et al. [14] failed to identify any difference among OPCAB and ONCAB/BH groups, in regard to preoperative patients profile and risk factors. Furthermore, the mean LVEF and the predicted risk for surgical intervention according to EuroSCORE were comparable between the two surgical approaches. On the contrary, the most notable characteristic of the subgroup of patients who underwent ONCAB/BH procedures at our institution was their severity of disease. They presented with severely deteriorated LVEF, poor clinical status as reflected by the EuroSCORE II, symptoms of congestive heart failure (NYHA class III/IV), and comorbidities such as renal impairment and cerebrovascular disease. Moreover, 41% of the cases in ONCAB/BH group were operated in an emergency setting.

A novel finding compared to other contemporary surgical series in high-risk patients was that the preoperative physiological reserve assessed by EuroSCORE II was identified as a powerful predictor for appraising the type of surgical approach that could be applied in beating heart revascularization [13, 14]. The unquestionable prognostic performance of EuroSCORE II is reinforced by the fact that takes into account the majority of patient-, cardiac-, and operation-related risk factors, already assessed as preoperative risk factors in our study group.

Furthermore, it has been documented that severe LV failure represents an unfavorable prognostic sign after CABG [5, 6]. Albeit there are data supporting that LV function is better preserved with an off-pump technique, limited preoperative cardiac performance incurs higher incidence of noteworthy hemodynamic derangement induced by the extensive mobilization and manipulation of the heart during off-pump CABG; hence, the theoretical need exists for mechanical support from a CPB circuit [4, 6]. This was our case, where LVEF presented a notable ability in identifying patients with an increased likelihood of being subjected to ONCAB/BH approach. This unplanned conversion to on-pump beating heart CABG frequently implicates postoperative course and final prognosis [11].

Clinical outcome is also significantly affected by circumstances encountered postoperatively in the ICU. Considering the poorer clinical status upon presentation of our ONCAB/BH cases compared with OPCAB ones, it is not surprising that these patients had a stormy postoperative ICU course. This was reflected by the higher incidence of LCOS development, need for inotropic support or IABP to maintain hemodynamic stability, sustained ventricular arrythmias or postoperative myocardial infarction, multiorgan system failure, and acute renal function impairment, which have already been identified as the principal complications in patients with poor LVEF undergoing coronary revascularization, influencing adversely both operative mortality and length of ICU stay [3–5, 13, 14]. Interestingly, the higher severity of acute illness upon ICU admission, and multiorgan impairment during the course of ICU stay (assessed by APACHE II and MaxSOFA score, resp.) emerged as potent indicators differentiating the two studied beating heart CABG techniques, further supporting the potential of a look at the evolution of patient risk during the treatment process. Since there are no other reports evaluating EuroSCORE II with SOFA score in this defined subset of high-risk cardiac surgery population, our results concerning these risk algorithms could not be thoroughly evaluated.

Albeit the use of ONCAB/BH technique among high risk patients is considered beneficial in terms of elimination of serious manipulations, preservation of native coronary perfusion, and perioperative myocardial metabolic function enhancement, from our data an augmented incidence of myocardial infarction among ONCAB/BH patients (11%) occurred [15]. This finding illustrates that the manipulation of the heart even though being supported by the bypass machine could still have adverse consequences on myocardial performance. This is in accordance with previous findings reporting an augmented incidence of new irreversible myocardial injury among patients with normal and compromised ventricular performance, who were subjected to ONCAB/BH compared with OPCAB technique [10, 14].

Several investigators ascertained that major postoperative complications such as sustained arrythmias, LCOS development, multiorgan failure, postoperative renal insufficiency, and length of intubation influence adversely time to ICU discharge [4, 12, 14]. In regard to early morbidity in our entire cohort (27.3%), which was assessed by prolongation of ICU stay for more than 48 hours, it was comparable to that previously reported in similar clinical settings [1–3, 5, 14, 16]. The incidence of morbidity recorded among the two studied beating heart approaches was affected by on-pump technique in a rather unfavorable manner (42.2% versus 23.4% in off-pump). Albeit the incidence of total major morbidity reported by Darwazah et al. [14] was higher among ONCAB/BH patients, the difference did not reach statistical significance. This discrepancy to our findings could probably be attributed to the fact that they failed to identify any notable differences in the perioperative characteristics of their study population. It seems that ONCAB/BH technique could not ameliorate the complications encountered with the use of CPB and effectively modify its negative impact in high-risk populations. Regarding this issue, OPCAB still remains advantageous.

Many recent studies have reported excellent results for CABG on the beating heart in patients with limited cardiac reserve, further reinforced by the mortality recorded in our study group (3.7%) [12–16]. In our study population, the ONCAB/BH procedures presented an increased (although not statistically significant) mortality rate compared with the OPCAB ones (6.6% versus 2.9%, resp.). The divergence in the reported mortality rate among patients with low LVEF undergoing ONCAB/BH (ranging from 2.6% to 6% and 8%) could be attributed to the difference in selection criteria, involving not only the degree of LVEF deterioration, but the coexistence of risk factors such as acute myocardial infarction, emergency or urgent setting, LCOS, or hemodialysis for renal function support [9, 11, 13, 14, 16, 17]. Taking into consideration all the aforementioned aspects, the relatively high operative mortality rate in our ONCAB/BH population could possibly be addressed to the poorer clinical status upon presentation and the more implicated ICU stay course, which both seem to confer an adverse impact on final prognosis. In a selected group of high-risk patients who underwent emergency multiple CABG using the on-pump beating heart technique and presented with similar pre- and postoperative clinical characteristics to our ONCAB/BH group, the reported in-hospital mortality was 8% [13]. This clearly underlines the importance of associated other risk factors affecting mortality beside impaired LVEF.

The authors acknowledge some limitations of the present study. The retrospective nature of this study and the assessment only of early-term morbidity and mortality in this subset of cardiac surgical patients, in conjunction to single-center study, limit the usefulness of our results. Long-term randomized controlled trials regarding OPCAB and ONCAB/BH surgery involving a larger number of participants could help clarify the parameters that impose assignment in each approach, the benefit of each procedure, and the risk factors involved in unfavourable outcome.

In conclusion, our findings indicate that patients with limited cardiac reserve undergoing isolated myocardial revascularization with the ONCAB/BH approach are subjected to more preoperative comorbidities and implicated ICU stay than their OPCAB counterparts. Furthermore, in this defined subset, the utilization of LVEF estimation and clinical status assessed by the EuroSCORE II seems to serve as discriminative predictors of intraoperative conversion from off-pump to on-pump beating heart technique, which in turn influences adversely only short-term morbidity, since operative mortality remains unaffected.Further studies are needed to delineate the clinical context of which the optimal clinical benefit might occur from the two alternatives of beating heart revascularization applied in ischemic cardiomyopathy.

## Figures and Tables

**Table 1:** Baseline and intraoperative risk factors.

Variable	OPCAB	ONCAB/BH	*P* value
Patient number	171	45	
Age (years)	64.2 ± 8	63.3 ± 8	0.514
Males	143 (84)	35 (78)	0.356
EuroSCORE II	1.7 ± 1.2	3.9 ± 1.8	0.000
NYHA class III/IV	113 (66)	41 (91)	0.001
LVEF (%)	34.8 ± 6	28.8 ± 8	0.000
Hypertension	87 (51)	22 (49)	0.768
Diabetes mellitus	60 (35)	19 (42)	0.396
Peripheral vascular disease	14 (8)	7 (15)	0.169
Cerebral vascular disease	5 (3)	6 (13)	0.006
Renal dysfunction	17 (10)	14 (31)	0.001
COPD	56 (33)	16 (35)	0.761
Acute presentation	3 (1.7)	18 (41)	0.000

Data are expressed as mean (±SD) or as counts (percentage).

OPCAB: off-pump coronary artery bypass grafting; ONCAB/BH: on-pump—beating heart coronary artery bypass grafting; EuroSCORE II: European System for Cardiac Operative Risk Evaluation II; NYHA: New York Heart Association; LVEF: left ventricular ejection fraction; COPD: chronic obstructive pulmonary disease; ONCABG/BH: on-pump coronary artery bypass grafting/beating heart.

**Table 2:** Postoperative risk factors, morbidity, and mortality.

Variable	OPCAB	ONCAB/BH	*P* value
Patient number	171	45	
APACHE II	12.9 ± 4	16.8 ± 6	0.001
SOFA score	4.1 ± 2.5	5.3 ± 3.6	0.041
LCOS	25 (15)	14 (31)	0.013
Use of inotropes	66 (38)	26 (58)	0.025
Use IABP	5 (3)	6 (13)	0.017
AMI	3 (2.3)	5 (11)	0.025
Arrythmias	49 (29)	19 (42)	0.115
AKI	8 (5)	7 (15)	0.019
Haemodialysis support	2 (1.5)	1 (2)	0.564
Respiratory complications	52 (30)	12 (28)	0.714
CNS dysfunction	12 (7)	5 (11)	0.482
Stroke	0	1 (2.2)	0.339
Reoperation	1 (0.7)	4 (9)	0.012
Mechanical ventilation (days)	1.02 ± 2	2.7 ± 6.4	0.045
ICU stay (days)	2.4 ± 4.4	5.1 ± 9.3	0.001
30-day mortality	5 (2.9)	3 (6.6)	0.224

Data are expressed as mean (±SD) or as counts (percentage).

OPCAB: off-pump coronary artery bypass grafting; ONCAB/BH: on-pump—beating heart coronary artery bypass grafting; APACHE II: Acute Physiology and Chronic Health Evaluation II; SOFA: System Organ Failure Assessment; LCOS: low cardiac output syndrome; IABP: intra-aortic balloon pump; AMI: acute myocardial infarction; AKI: acute kidney injury; CNS: central nervous system; ICU: intensive care unit.
